# High-mobility hydrogenated polycrystalline In_2_O_3_ (In_2_O_3_:H) thin-film transistors

**DOI:** 10.1038/s41467-022-28480-9

**Published:** 2022-02-28

**Authors:** Yusaku Magari, Taiki Kataoka, Wenchang Yeh, Mamoru Furuta

**Affiliations:** 1 Graduate School of Natural Science and Technology, Shimane University, Matsue, Shimane 690-8504 Japan; 2 School of Environmental Science and Engineering, Kochi University of Technology, Kami, Kochi 782-8502 Japan; 3 Center for Nanotechnology, Research Institute, Kochi University of Technology, Kami, Kochi 782-8502 Japan

**Keywords:** Electronic devices, Sensors and biosensors

## Abstract

Oxide semiconductors have been extensively studied as active channel layers of thin-film transistors (TFTs) for electronic applications. However, the field-effect mobility (*μ*_FE_) of oxide TFTs is not sufficiently high to compete with that of low-temperature-processed polycrystalline-Si TFTs (50–100 cm^2^V^−1^s^−1^). Here, we propose a simple process to obtain high-performance TFTs, namely hydrogenated polycrystalline In_2_O_3_ (In_2_O_3_:H) TFTs grown via the low-temperature solid-phase crystallization (SPC) process. In_2_O_3_:H TFTs fabricated at 300 °C exhibit superior switching properties with *µ*_FE_ = 139.2 cm^2^V^−1^s^−1^, a subthreshold swing of 0.19 Vdec^−1^, and a threshold voltage of 0.2 V. The hydrogen introduced during sputter deposition plays an important role in enlarging the grain size and decreasing the subgap defects in SPC-prepared In_2_O_3_:H. The proposed method does not require any additional expensive equipment and/or change in the conventional oxide TFT fabrication process. We believe these SPC-grown In_2_O_3_:H TFTs have a great potential for use in future transparent or flexible electronics applications.

## Abstract (web summary)

The field-effect mobility of oxide semiconductor thin-film transistors is not sufficiently high compared to silicon thin-film transistors. Magari et al. use a low-temperature solid-phase crystallization process to fabricate In_2_O_3_ thin-film transistors with mobility comparable to silicon counterparts.

## Introduction

Wide-bandgap oxide semiconductors (OSs) have been extensively studied as active channel layers of thin-film transistors (TFTs) for next-generation flat-panel displays^1,2^, nonvolatile memories^3^, inverters^4^, various sensors^5,6^, Schottky devices^7,8^, and so on. Among OSs, amorphous In–Ga–Zn–O (a-IGZO) TFTs have now become the backplane standard for active-matrix liquid-crystal displays (AMLCDs) and active-matrix organic light-emitting diode (AMOLED) displays because of their reasonable field-effect mobility (*μ*_FE_) of over 10 cm^2^ V^−1^ s^−1^, extremely low leakage current, low process temperature (<350 °C), and large-area scalability^9,10^. Although the *μ*_FE_ value of a-IGZO TFTs is more than ten times higher than that of hydrogenated amorphous Si (a-Si:H) TFTs (<1 cm^2^ V^−1^ s^−1^), it is not sufficiently high to compete with that of low-temperature-processed polycrystalline-Si (LTPS) TFTs (50–100 cm^2^ V^−1^ s^−1^)^11^. The main disadvantages of LTPS TFTs are a relatively high process temperature (450–550 °C) and an expensive crystallization process. The high *μ*_FE_ values of OS TFTs mean these devices could be used in fields that have been dominated by LTPS TFTs and in transparent and flexible devices that are incompatible with Si. Numerous types of approaches to enhance the *μ*_FE_ value of OS TFTs have been investigated, including cation composition^12,13^, multiple channel structures^14,15^, dual-gate architecture^16,17^, metal capping layer structures^18,19^, post treatment^20,21^, and their combination. Among these, cation composition control is the most promising method. It requires no extra complex process for integrating OS TFTs. In-rich OSs have been studied extensively. The large spatial spread of the In 5*s* orbital with a large overlap can provide a facile electron transport path with a low electron effective mass^22^. However, undoped In_2_O_3_ films exhibit a high background electron concentration (10^19^–10^21^ cm^−3^)^23,24^, which is attributable to the presence of native defects, such as oxygen vacancies, making it difficult to control the threshold voltage of the TFTs^25,26^. To suppress the carrier concentration in In_2_O_3_, elements such as Ga, Hf, Si, Al, and W were added, which have large bond dissociation energies with oxygen^12^. Many AOS TFTs have been explored with multicomponent oxide semiconductors, such as In–W–Zn–O^27^, Al–In–Sn–Zn–O^28^, and In–Ga–Zn–Sn–O^29^. However, multicomponent oxides complicate the composition control of the deposited film. Moreover, multimetal cations cause potential fluctuation near the conduction band minimum, which might hinder electron transport^30^.

Recently, crystalline OSs have been proposed to enhance the carrier mobility because the disorder-induced subgap states can be suppressed via lattice ordering. Yang et al. reported a *μ*_FE_ value of 60.7 cm^2^ V^−1^ s^−1^ for a TFT obtained using polycrystalline In–Ga–O annealed at 700 °C^31^. Although high annealing temperatures result in better electrical properties of the oxide active channel layer, such high temperatures are unsuitable for device application on glass or plastic substrates. Our group reported a *μ*_FE_ value of 50.6 cm^2^ V^−1^ s^−1^ for a TFT obtained using hydrogenated polycrystalline In–Ga–O formed via solid-phase crystallization (SPC) at 300 °C^32^.

This study proposes a simple material and a simple process to obtain high-performance TFTs, namely hydrogenated polycrystalline In_2_O_3_ (In_2_O_3_:H) TFTs grown via the low-temperature SPC process. In_2_O_3_:H TFTs fabricated at 300 °C exhibit superior switching properties with *µ*_FE_ = 139.2 cm^2^ V^−1^ s^−1^, a subthreshold swing (*SS*) of 0.19 V dec^−1^, and a threshold voltage (*V*_th_) of 0.2 V. The hydrogen introduced during sputter deposition plays an important role in enlarging the grain size and decreasing the subgap defects in SPC-prepared In_2_O_3_:H. The proposed method has great potential for future transparent or flexible electronics applications.

## Results

### Structural properties of the In_2_O_3_ and In_2_O_3_:H films

**Fig. 1:**
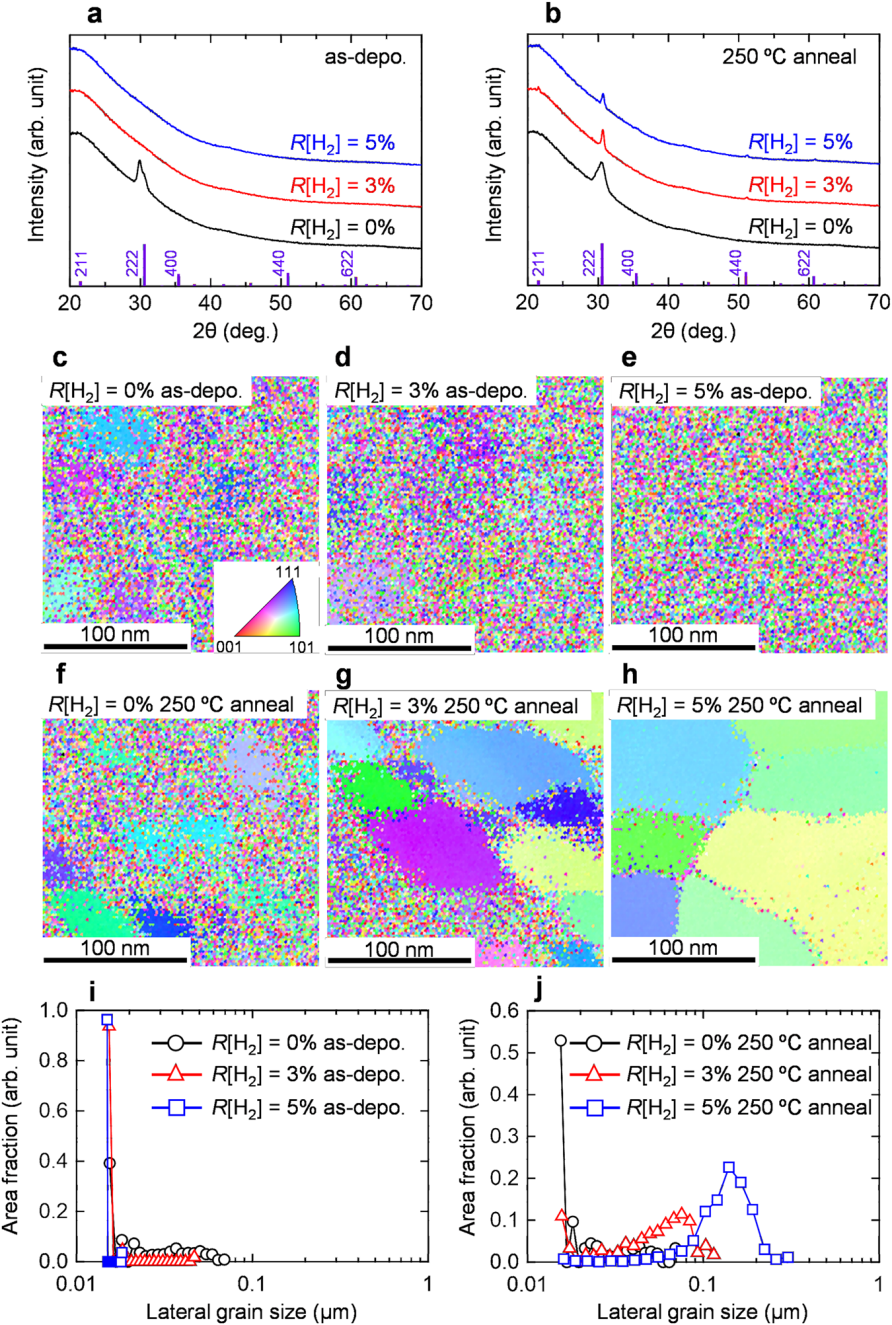
Structural properties of the In_2_O_3_ and In_2_O_3_:H films. XRD patterns of the In_2_O_3_ and In_2_O_3_:H films deposited at different *R*[H_2_] values and at a constant *R*[O_2_] value of 1% **a** before and **b** after annealing at 250 °C in N_2_. EBSD images of the In_2_O_3_ and In_2_O_3_:H films deposited at different *R*[H_2_] values **c**–**e** before and **f**–**h** after annealing at 250 °C in N_2_. Area fraction of each grain size obtained from the In_2_O_3_ and In_2_O_3_:H films deposited at different *R*[H_2_] values **i** before and **j** after annealing at 250 °C in N_2_.

Figures 1a, b show the XRD patterns of the 50-nm-thick In_2_O_3_ and In_2_O_3_:H films deposited at various *R*[H_2_] values and at a constant *R*[O_2_] of 1%. For the as-deposited films (Fig. 1a), the In_2_O_3_ film without H_2_ introduction exhibited a clear crystalline nature with the (222) preferred orientation of the cubic bixbyite In_2_O_3_ crystal. There was no noticeable peak for the In_2_O_3_:H films deposited at *R*[H_2_] values of 3 and 5%. This result indicates that H_2_ addition suppresses the growth of crystallites during deposition. After annealing at 250 °C in nitrogen for 1 h, the amorphous phase of In_2_O_3_:H changed to the crystalline one with the (222) preferred orientation. The angles of the diffracted peaks are in good agreement with the In_2_O_3_ powder data (ICSD code: 14388). Moreover, the crystallized films exhibited smaller full-width at half-maximum values of the (222) reflection than the film deposited without hydrogen introduction, indicating larger crystallite sizes and smaller strains in the In_2_O_3_:H films.

Figure 1c–h depict the EBSD images along the normal direction for the In_2_O_3_ and In_2_O_3_:H films with and without annealing at 250 °C in nitrogen. For the as-deposited films (Fig. 1c–e), a randomly oriented small grain structure embedded in the amorphous matrix can be observed in the In_2_O_3_ film without H_2_ introduction. The grain structure disappeared upon increasing the *R*[H_2_] value to 5%. In contrast, a huge grain structure appeared for In_2_O_3_:H deposited at *R*[H_2_] = 5% after annealing at 250 °C (Fig. 1h), indicating SPC occurrence. This is consistent with the results of the XRD analysis shown in Fig. 1a, b. The corresponding area fractions of each lateral grain size are shown in Fig. 1i, j. The detected minimum grain size is around 15 nm, which is limited by the electron beam size of the EBSD measurements. For the as-deposited films (Fig. 1i), all films showed the maximum area fraction for a grain size of 15 nm; however, a small proportion of the area fraction with a grain size of ~70 nm was detected in the In_2_O_3_ film, indicating nuclei in the as-deposited film. After annealing at 250 °C (Fig. 1j), the peak of the area fraction shifted toward a larger grain size as *R*[H_2_] increased, and the In_2_O_3_:H film deposited at *R*[H_2_] = 5% showed a maximum area fraction of 23% at a grain size of 140 nm. Furthermore, as *R*[H_2_] increased from 0 to 5%, the area fraction of the minimum grain size below 15 nm significantly decreased, and only a few small grains were in between the large grains, as shown in Fig. 1h. Similar results were observed for films annealed at 250 °C in ambient air (shown in Supplementary Fig. 1). The EBSD results show that the nuclei density in the as-deposited film was suppressed by introducing hydrogen during sputtering. Because of the reduction in the nuclei density in the initial In_2_O_3_:H film, the grain size of the In_2_O_3_:H film could be enlarged through SPC. Thus, the XRD and EBSD results indicate that controlling the crystallinity and nuclei density in the as-deposited film are key factors to achieve high-quality In_2_O_3_:H films.

### Electrical and optical properties of the In_2_O_3_ and In_2_O_3_:H films

**Fig. 2:**
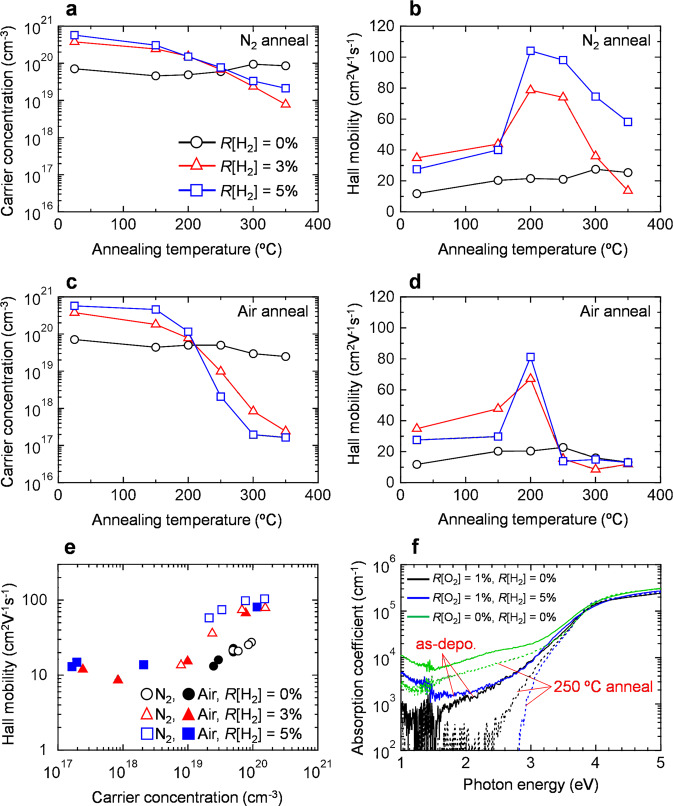
Electrical and optical properties of the In_2_O_3_ and In_2_O_3_:H films. **a** *N*_e_ and **b** *µ*_H_ of In_2_O_3_ and In_2_O_3_:H deposited at different *R*[H_2_] values as a function of the annealing temperature in N_2_. **c** *N*_e_ and **d** *µ*_H_ of In_2_O_3_ and In_2_O_3_:H deposited at different *R*[H_2_] values as a function of the annealing temperature in ambient air. **e** The relationship between *µ*_H_ and *N*_e_ for the films annealed at *T*_ann_ > 200 °C in N_2_ and ambient air. **f** Optical absorption spectra of the 50-nm-thick In_2_O_3_ and In_2_O_3_:H films deposited at different *R*[H_2_] values before and after annealing at 250 °C in ambient air. In_2_O_3_ films deposited without oxygen and hydrogen are also shown for comparison.

Figure 2a–d show the carrier concentration (*N*_e_) and Hall mobility (*µ*_H_) of the 50-nm-thick In_2_O_3_ and In_2_O_3_:H films deposited at various *R*[H_2_] values as a function of the annealing temperature (*T*_ann_). Koida et al. reported that both the *N*_e_ and *µ*_H_ of the SPC-prepared In_2_O_3_:H films decrease dramatically for the films annealed in vacuum at *T*_ann_ > 400 °C due to the desorption of the H_2_O and H_2_ gases from the films and additional microscopic defects inside the grains^33^. The electrical properties of OSs are strongly affected by the annealing atmosphere^34^; thus, annealing treatments at temperatures ranging from 150 to 350 °C in nitrogen and ambient air were examined. For the as-deposited films, *N*_e_ increased from 7.1 × 10^19^ to 5.7 × 10^20^ cm^−3^ upon increasing *R*[H_2_] from 0 to 5%, as shown in Fig. 2a. Since hydrogen acts as a shallow donor in In_2_O_3_^35^, the increase in the *N*_e_ of the as-deposited In_2_O_3_:H film upon increasing *R*[H_2_] is attributable to hydrogen doping effects. The In_2_O_3_ film deposited without hydrogen introduction exhibited an almost constant *N*_e_ value over the whole range of investigated *T*_ann_ values irrespective of the annealing atmosphere, as shown in Fig. 2a, c. In contrast, there was a strong dependence of *N*_e_ on the annealing atmosphere for the In_2_O_3_:H films. The *N*_e_ of the In_2_O_3_:H film annealed in N_2_ gradually decreased with increasing *T*_ann_ (Fig. 2a), whereas the *N*_e_ of the In_2_O_3_:H film annealed in air rapidly decreased for *T*_ann_ > 200 °C (Fig. 2c). In addition, the *N*_e_ reduction was remarkable in the In_2_O_3_:H film deposited at a higher *R*[H_2_] value. As a result, an appropriate *N*_e_ value of 2.0 × 10^17^ cm^−3^ for TFT fabrication was obtained at *T*_ann_ = 300 °C for the In_2_O_3_:H film deposited at *R*[H_2_] = 5%; this *N*_e_ value is over two orders of magnitude lower than that of the In_2_O_3_ film deposited without hydrogen introduction (3.0 × 10^19^ cm^−3^). Such a large decrease in the *N*_e_ value of the In_2_O_3_:H films has not been reported before. Although adding H_2_ induced the formation of free carriers in the as-deposited films, the *N*_e_ of the films could be reduced via the relatively low-temperature SPC process and became comparable to that of single-crystalline epitaxial In_2_O_3_ films deposited at 650 °C (~1 × 10^17^ cm^−3^)^36^.

Regarding the Hall mobility of the films, In_2_O_3_ without hydrogen introduction exhibited an almost constant *µ*_H_ value over the entire range of investigated *T*_ann_ values irrespective of the annealing atmosphere, as shown in Fig. 2b, c. Upon annealing in N_2_ at *T*_ann_ = 200 °C, the *µ*_H_ of In_2_O_3_:H increased to 78.6 cm^2^ V^−1^ s^−1^ at *R*[H_2_] = 3% and 104.0 cm^2^ V^−1^ s^−1^ at *R*[H_2_] = 5%, indicating that the SPC started at a *T*_ann_ value between 150 and 200 °C. Furthermore, the increased *µ*_H_ is attributable to the increased grain size, as shown in Fig. 1f–h. As *T*_ann_ increased, the *µ*_H_ of In_2_O_3_:H gradually decreased (Fig. 2b). Upon annealing in air (Fig. 2d), the maximum *µ*_H_ of In_2_O_3_:H decreased slightly to 67.1 cm^2^ V^−1^ s^−1^ at *R*[H_2_] = 3% and 81.2 cm^2^ V^−1^ s^−1^ at *R*[H_2_] = 5%, and the decrease in *µ*_H_ for *T*_ann_ > 250 °C was confirmed.

To understand the transport properties of the In_2_O_3_:H films after SPC, the relationship between *µ*_H_ and *N*_e_ for the films annealed in the range of temperatures between 200 °C and 350 °C in N_2_ and ambient air was summarized, as shown in Fig. 2e. The *N*_e_ of the In_2_O_3_:H film could be controlled by up to three orders of magnitude. Moreover, for the *N*_e_ range between 10^19^ and 10^20^ cm^−3^, *µ*_H_ increases with increasing *R*[H_2_], which is attributable to the suppression of grain boundary scattering due to the increasing grain size. For all the films, *µ*_H_ increased with increasing *N*_e_. In general, the grain boundaries have a small impact on *µ*_H_ in transparent conductive oxides with high *N*_e_ (>10^20^ cm^−3^) because electrons can tunnel through the narrow width (<1 nm) of the grain barriers at high *N*_e_ values (>10^20^ cm^−3^). However, grain boundary scattering is a dominant factor that limits the *µ*_H_ in films with lower *N*_e_^37^. Thus, the observed decrease in *µ*_H_ with decreasing *N*_e_ (Fig. 2e) is due to grain boundary scattering. Although the *µ*_H_ of In_2_O_3_:H decreased for *N*_e_ < 10^19^ cm^−3^, carriers (with number in the range of 10^19^–10^20^ cm^−3^) will be generated at the In_2_O_3_:H/gate insulator interface of the TFTs when applying a voltage to the gate^15^; thus, a high *µ*_FE_ can be expected in In_2_O_3_:H TFTs.

Figure 2f shows the optical absorption (*α*) spectra of the In_2_O_3_ and In_2_O_3_:H films before and after annealing at 250 °C in ambient air. The green line represents the In_2_O_3_ film deposited without oxygen and hydrogen (only Ar gas), which is shown for comparison. The spectral features that arise as the photon energy exceeds 2.9 eV are due to the absorption associated with the interband transition in In_2_O_3_, whereas the features that arise when the photon energy is below 1.5 eV are due to free carrier absorption. The absorption in the subgap region (<2.9 eV) dropped as *R*[O_2_] increased from 0 to 1%, suggesting that oxygen deficiencies, which give rise to subgap defects, are compensated when sputtering in an oxidizing atmosphere. When hydrogen is added during sputter deposition, the absorption is enhanced in the subgap region for the as-deposited films, especially in the photon energy region below 1.5 eV, indicating that free electron absorption is increased due to the hydrogen doping effect. On the other hand, after annealing at 250 °C in ambient air (dashed line), the absorption across the subgap of the In_2_O_3_:H film decreased significantly and was lower than that of the In_2_O_3_ film. Since subgap defects are generated from native defects, such as oxygen vacancies, as described above, it can be inferred that oxygen vacancies were efficiently reduced in In_2_O_3_:H via SPC in ambient air. This result is consistent with the Hall effect measurements, where it was found that *N*_e_ decreased from 5.7 × 10^20^ to 2.0 × 10^18^ cm^−3^ upon annealing in air at 250 °C, as shown in Fig. 2c. The structural, electrical, and optical properties of the In_2_O_3_:H films show that the hydrogen introduced during sputter deposition plays an important role in enlarging the grain size and decreasing the subgap defects after SPC, increasing *µ*_H_ and decreasing *N*_e_. However, a more detailed study will be necessary to carry out quantitative evaluations of the carrier generation and scattering in the SPC-prepared In_2_O_3_:H films.

### In_2_O_3_:H TFT characteristics

**Fig. 3:**
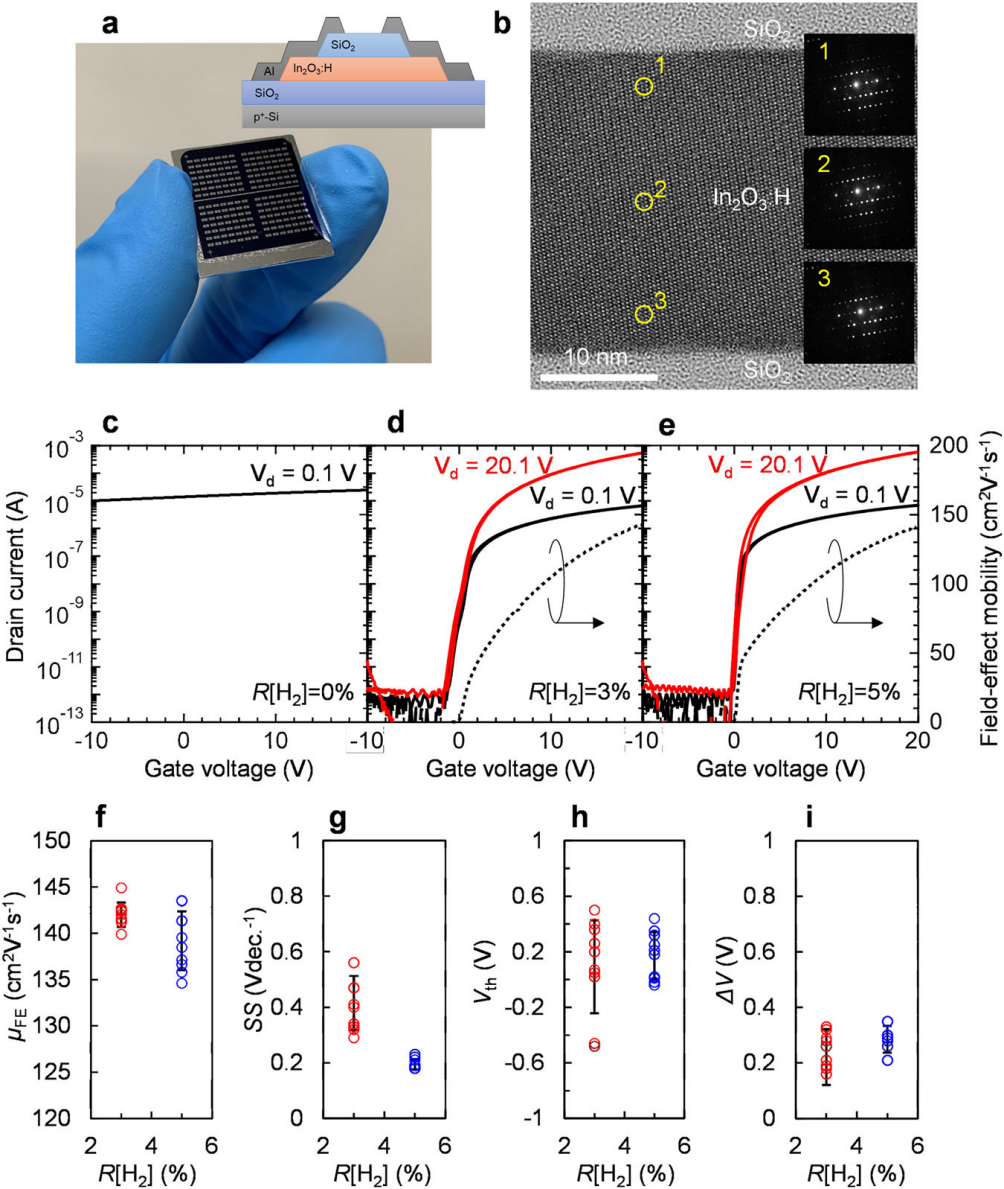
In_2_O_3_:H TFT characteristics. **a** Photograph and schematic cross-sectional view of the SPC-prepared In_2_O_3_:H TFTs. **b** HRTEM image and corresponding SAED pattern of the active layer of the TFT with In_2_O_3_:H channel deposited at *R*[H_2_] = 5%. **c**–**e** Typical transfer characteristics of the TFTs with In_2_O_3_ and In_2_O_3_:H channels deposited at various *R*[H_2_] values. Variations of **f** *µ*_FE_, **g** SS, **h** *V*_th_, and **i** Δ*V* evaluated from ten TFTs.

Figure 3a shows the picture and schematic cross-sectional view of the fabricated In_2_O_3_:H TFT. All TFTs were fabricated using annealing in ambient air at 300 °C. Figure 3c–e show typical transfer characteristics of the TFTs with In_2_O_3_ and In_2_O_3_:H channels deposited at various *R*[H_2_] values. The variations of *µ*_FE_, subthreshold swing (SS), threshold voltage (*V*_th_), and hysteresis (Δ*V*) were evaluated from ten TFTs on the same substrate. The *µ*_FE_ was calculated from the linear transfer characteristics as *µ*_FE_ = *Lg*_m_/(WC_ox_*V*_ds_) at *V*_ds_ = 0.1 V, where *g*_m_ is the transconductance, *C*_ox_ is the oxide capacitance of the gate insulator, and *V*_ds_ is the drain voltage. *V*_th_ was defined by gate voltage (*V*_gs_) at drain current (*I*_ds_) of 1 nA, and *SS* was extracted from *V*_gs_, which required an increase in the *I*_ds_ from 10 to 100 pA. The In_2_O_3_ TFT without H_2_ introduction (Fig. 3c) did not exhibit any switching (conductive behavior). By contrast, the In_2_O_3_:H TFT deposited at *R*[H_2_] = 5% exhibited a switching with an extremely high *µ*_FE_ of 139.2 cm^2^ V^−1^ s^−1^, a SS of 0.19 V dec^−1^, a *V*_th_ of 0.2 V, and a Δ*V* of 0.3 V as shown in Table 1. The TFT with In_2_O_3_:H deposited at *R*[H_2_] = 5% showed a slightly small *SS* value (0.19 V dec^−1^) compared with that of the TFT with In_2_O_3_:H deposited at *R*[H_2_] = 3% (0.41 V dec^−1^). The subgap density of states (*D*_sg_) at the Fermi level was calculated as SS = d*V*_gs_/dlog*I*_ds_ = ln10 *k*_B_*T*/*e* (1 + *eD*_sg_/*C*_ox_), where *k*_B_ is Boltzmann’s constant and *e* is the elementary electric charge^10^. The *D*_sg_ decreased from 1.3 × 10^12^ to 4.7 × 10^11^ cm^−2^ eV^−1^ by increasing the *R*[H_2_] value from 3 to 5%. This result suggests that the disorder-originated subgap defect states near the conduction band minimum can be reduced in the In_2_O_3_:H channel deposited at *R*[H_2_] = 5%, which is confirmed by the optical measurements of the films shown in Fig. 2f.

**Table 1:** Summary of the TFT properties.

R[O_2_] (%)	R[H_2_] (%)	*µ*_FE_ (cm^2^ V^−1^ s^−1^)	*SS* (V dec^−1^)	*V*_th_ (V)	Δ*V* (V)	*D*_sg_ (cm^−2^ eV^−1^)
1	0	–	–	–		–
1	3	142.0 (1.2)	0.41 (0.09)	0.1 (0.3)	0.2 (0.1)	1.3 × 10^12^
1	5	139.2 (3.0)	0.19 (0.02)	0.2 (0.2)	0.3 (0.1)	4.7 × 10^11^

The average values and standard deviations (*σ*) of the characteristics of ten TFTs on the same substrate. *σ* are shown in parentheses.

The resulting transfer performance of the SPC-prepared In_2_O_3_:H TFT (*R*[H_2_] = 5%) was superior to that of previously reported oxide-based TFTs^38^. Although the *µ*_H_ of the In_2_O_3_:H (*R*[H_2_] = 5%) films decreased to 14.9 cm^2^ V^−1^ s^−1^ with *N*_e_ of 2.0 × 10^17^ cm^−3^ by annealing at 300 °C as shown in Fig. 2c, extremely high *µ*_FE_ was obtained from the TFTs because a large number of carriers (10^19^–10^20^ cm^−3^) was generated at the In_2_O_3_:H/gate insulator interface when applying a voltage to the gate, which allows electrons to tunnel through the narrow width (<1 nm) of the grain barriers at high *N*_e_ values. The high *µ*_FE_ and steep *SS* of the In_2_O_3_:H TFTs can be attributed to the high crystallinity of In_2_O_3_:H, especially near the In_2_O_3_:H/SiO_2_ gate insulator interface.

Figure 3b shows a cross-sectional conventional bright-field HRTEM image and selective area electron diffraction (SAED) pattern obtained from the SPC-prepared In_2_O_3_:H TFT (*R*[H_2_] = 5%). A clear lattice image was observed over the entire thickness of the In_2_O_3_:H channel. Moreover, there was a single crystal-like diffraction pattern in the SAED pattern, even in the thin layers, roughly at a distance of 5 nm from the SiO_2_ gate insulator without detectable diffuse ring patterns, which would be attributable to an amorphous phase. This observation explains the high *µ*_FE_ of 139.2 cm^2^ V^−1^ s^−1^ in the In_2_O_3_:H TFTs, which is comparable to the *µ*_H_ of epitaxial single-crystal In_2_O_3_ films (~160 cm^2^ V^−1^ s^−1^)^39^. In addition, although In_2_O_3_:H is a polycrystalline film, the standard deviations (*σ*) of *µ*_FE_, *SS*, *V*_th_, and Δ*V* of the In_2_O_3_:H TFT (*R*[H_2_] = 5%) were 3.0 cm^2^ V^−1^ s^−1^, 0.02 V dec^−1^, 0.2 V, and 0.1 V, respectively, indicating high uniformity of the TFT characteristics (shown in Fig. 3f–i and Supplementary Fig. 2).

**Fig. 4:**
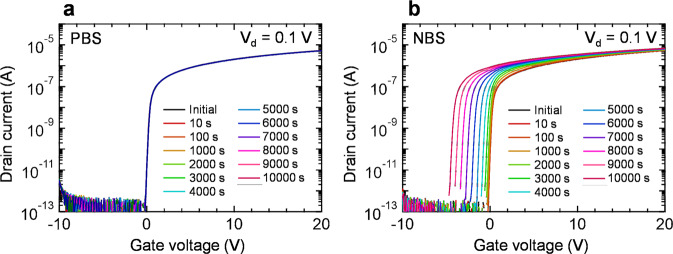
Reliability of the In_2_O_3_:H TFT. Changes in the transfer characteristics of the In_2_O_3_:H (*R*[H_2_] = 5%) TFT during the **a** PBS and **b** NBS tests. The *V*_gs_ values under the PBS and NBS tests were +20 and −20 V, respectively.

To investigate the reliability of the SPC-prepared In_2_O_3_:H TFT (*R*[H_2_] = 5%), positive-bias stress (PBS) and negative-bias stress (NBS) tests were carried out under a humidity of 50%. The gate stress voltages for the PBS and NBS were +20 and −20 V, respectively. Figure 4a, b show the changes in the transfer characteristics of the In_2_O_3_:H TFT during the PBS and NBS tests. The In_2_O_3_:H TFT showed no significant positive shift in *V*_th_ (only +0.02 V) under the PBS test, indicating the negligible interfacial trap states in the In_2_O_3_:H/SiO_2_ gate insulator interface as well as the high quality of the In_2_O_3_:H channel layer. In contrast, a large *V*_th_ shift of –4.4 V was observed for the NBS test, as shown in Fig. 4b. Furthermore, the *V*_th_ shift became more significant when the NBS test was conducted at a higher humidity of 70% (shown in Supplementary Fig. 3). Water molecules are coupled to the backchannel of the IGZO TFTs, and excess electrons are donated to the channel under NBS, resulting in a negative *V*_th_ shift^40,41^. Applying a passivation layer to the TFTs is effective in minimizing the influence of the atmospheric environment; however, hydrogen can diffuse into the channel through the passivation layer and increase the *N*_e_ of the channel^42^. Although the SiO_2_ passive layer was applied to the SPC-grown In_2_O_3_:H TFT, as shown in Fig. 3a, its protection ability was insufficient because the SiO_2_ film was deposited via sputtering at RT. Hence, it is believed that the reliability of In_2_O_3_:H TFTs can be improved by selecting the appropriate passivation layer, such as SiN, Al_2_O_3_, or Y_2_O_3_.

## Discussion

In this study, we demonstrate the high-performance polycrystalline In_2_O_3_:H TFTs using a low-temperature SPC process. To ensure the amorphous state of the as-deposited In_2_O_3_:H film, a moderate amount of H_2_ was introduced into the sputtering system during the In_2_O_3_:H deposition. The as-deposited amorphous In_2_O_3_:H film converted into a polycrystalline In_2_O_3_:H film with a grain size of around 140 nm via low-temperature SPC (at a temperature below 200 °C). As a result, a high *µ*_H_ of 104.0 cm^2^ V^−1^ s^−1^ was obtained for the In_2_O_3_:H film. This *µ*_H_ value is five times higher than that of the In_2_O_3_ film without H_2_ introduction during sputtering. Furthermore, the *N*_e_ of the SPC-grown In_2_O_3_:H film decreased significantly to 2.0 × 10^17^ cm^−3^ after annealing at 300 °C in ambient air; this *N*_e_ value is two orders of magnitude lower than that of the In_2_O_3_ film without H_2_ introduction. Thus, introducing hydrogen during sputtering followed by annealing in ambient air is an effective method for improving both the crystallinity and the *N*_e_ value of In_2_O_3_:H films. The obtained In_2_O_3_:H film was employed as the channel of a TFT, and the resulting In_2_O_3_:H TFT exhibits an extremely high *µ*_FE_ of 139.2 cm^2^V^−1^s^−1^, an appropriate *V*_th_ of 0.2 V, and a small SS of 0.19 V dec^−1^. HRTEM analysis of the TFT revealed the high crystallinity of cubic bixbyite near the In_2_O_3_:H/SiO_2_ gate dielectric interface, which contributed to the high *µ*_FE_ of the TFT. The proposed method does not require any additional expensive equipment and/or change in the conventional oxide TFT fabrication process. Moreover, composition control of binary In_2_O_3_ films is easier than that of ternary and quaternary semiconductors. We believe that these SPC-grown In_2_O_3_:H TFTs are promising candidates for use in future transparent or flexible electronics applications.

## Methods

### Fabrication of In_2_O_3_:H TFTs

In_2_O_3_:H TFTs were fabricated on a heavily doped *p*-type Si substrate with a 100-nm-thick thermally grown SiO_2_. The doped *p*-type Si substrate and the SiO_2_ were used as the gate electrode and the gate insulator. The 30-nm-thick In_2_O_3_ and In_2_O_3_:H channels were deposited via pulsed direct-current (DC) magnetron sputtering without substrate heating from a ceramic In_2_O_3_ target using a mixture of Ar, O_2_, and H_2_ gases. The O_2_ and H_2_ gas flow ratios are *R*[O_2_] = O_2_/(Ar + O_2_ + H_2_) and *R*[H_2_] = H_2_/(Ar + O_2_ + H_2_), respectively. For the Ar + O_2_-sputtered In_2_O_3_ film, *R*[O_2_] was set to 1% without H_2_ introduction. For the Ar + O_2_ + H_2_-sputtered In_2_O_3_ film (In_2_O_3_:H), *R*[H_2_] varied from 3 to 5%, whereas *R*[O_2_] was fixed at 1%. The deposition pressure and DC power were maintained at 0.6 Pa and 50 W, respectively. The base pressure before gas introduction was below 6 × 10^−5^ Pa. The In_2_O_3_ and In_2_O_3_:H films were then annealed in ambient air at 300 °C for 1 h. After annealing, a 100-nm-thick SiO_2_ film was deposited via reactive sputtering without substrate heating. This film served as a passive layer. Subsequently, Al source/drain electrodes were deposited via sputtering. Finally, In_2_O_3_ and In_2_O_3_:H TFTs were annealed at 250 °C in ambient air for 1 h. The In_2_O_3_, SiO_2_, and Al films were deposited through a shadow mask. Both the channel length and the width were 300 µm.

### Characterization of the In_2_O_3_:H films and TFTs

Structural, electrical, and optical measurements were conducted on the 50-nm-thick In_2_O_3_ and In_2_O_3_:H films deposited on a synthetic quartz substrate. The films’ structural changes were evaluated through X-ray diffraction (XRD) (Philips corp., X’pert) with CuK*α* radiation and electron backscattering diffraction (EBSD) (EDAX-TSL Hikari High Speed EBSD Detector). The films’ carrier concentrations (*N*_e_) and Hall mobility (*µ*_H_) were determined via Hall effect measurements (Accent, HL5500PC) using the van der Pauw geometry at room temperature (RT). The films’ optical properties were measured via spectrophotometry (Hitachi, U-4100). The current–voltage characteristics were measured using a semiconductor parameter analyzer (Keysight, E5270B) at RT in the dark. High-resolution transmission electron microscopy (HRTEM) (JEOL, JSM-7001F) analysis was also conducted to observe the microstructure of the In_2_O_3_:H channel in the TFTs.

## Supplementary information

Supplementary Information

## Data Availability

The authors declare that the all the data supporting the finding of this study are available within this article and its Supplementary Information files and are available from the corresponding author on reasonable request.
