# Supplementary material for: High-mobility hydrogenated polycrystalline In2O3 (In2O3:H) thin-film transistors
Source: Nat Commun. 2022 Feb 28;13:1078. doi: 10.1038/s41467-022-28480-9 (PMC8885685; doi:10.1038/s41467-022-28480-9)
Supplement: Supplementary file 1 — Supplementary Information [file 41467_2022_28480_MOESM1_ESM.pdf]

Supplementary Information

High-Mobility Hydrogenated Polycrystalline In_2O_3 (In_2O_3:H) Thin-Film Transistors

Yusaku Magari[1*], Taiki Kataoka[2], Wenchang Yeh[1], and Mamoru Furuta[2, 3]

[1]Graduate School of Natural Science and Technology, Shimane University, Matsue, Shimane 690-8504, Japan

[2]School of Environmental Science and Engineering, Kochi University of Technology, Kami, Kochi 782-8502, Japan

[3]Center for Nanotechnology, Research Institute, Kochi University of Technology, Kami, Kochi 782-8502, Japan

*E-mail: magari.yusaku@riko.shimane-u.ac.jp

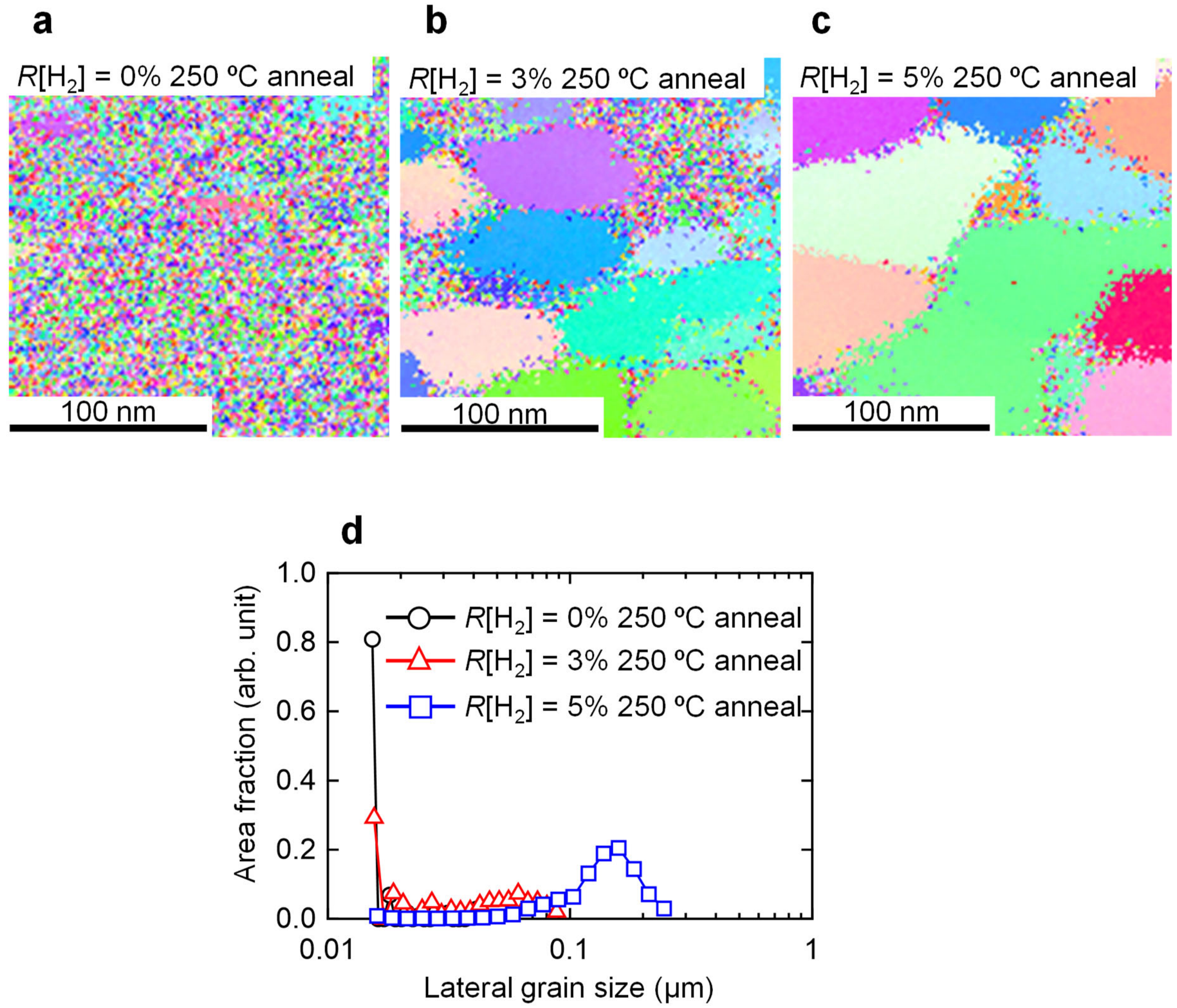

Supplementary Figure 1. (a–c) EBSD images of the In_2O_3 and In_2O_3:H films deposited at different R[H_2] values after annealing at 250 °C in ambient air. (d) Area fraction of each grain size obtained from the In_2O_3 and In_2O_3:H films deposited at different R[H_2] values after annealing at 250 °C in ambient air.

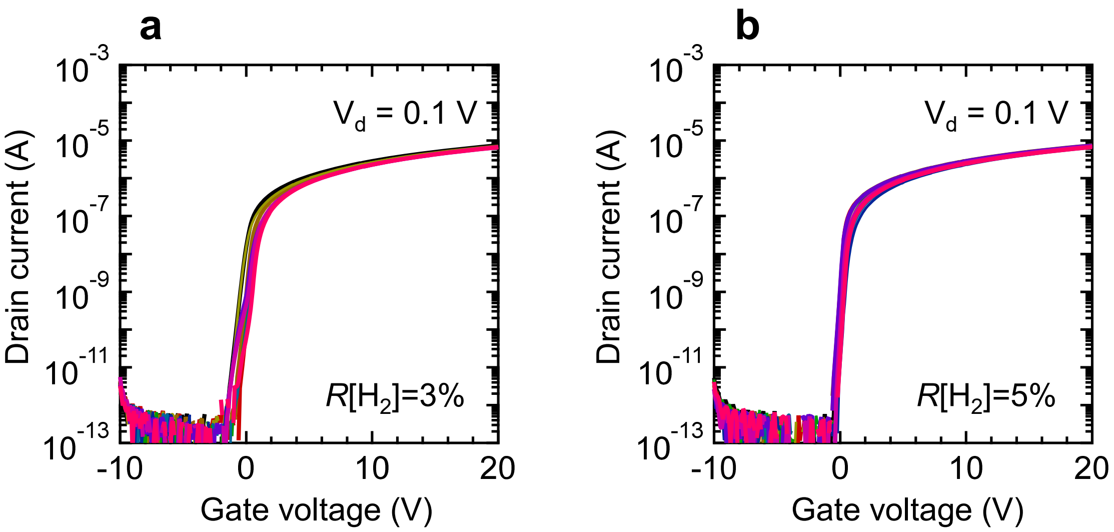

Supplementary Figure 2. (a, b) Variations of transfer characteristics of the In_2O_3:H TFTs with channels deposited at various $R[H_2]$ values. Ten TFTs on the same substrate were measured, respectively.

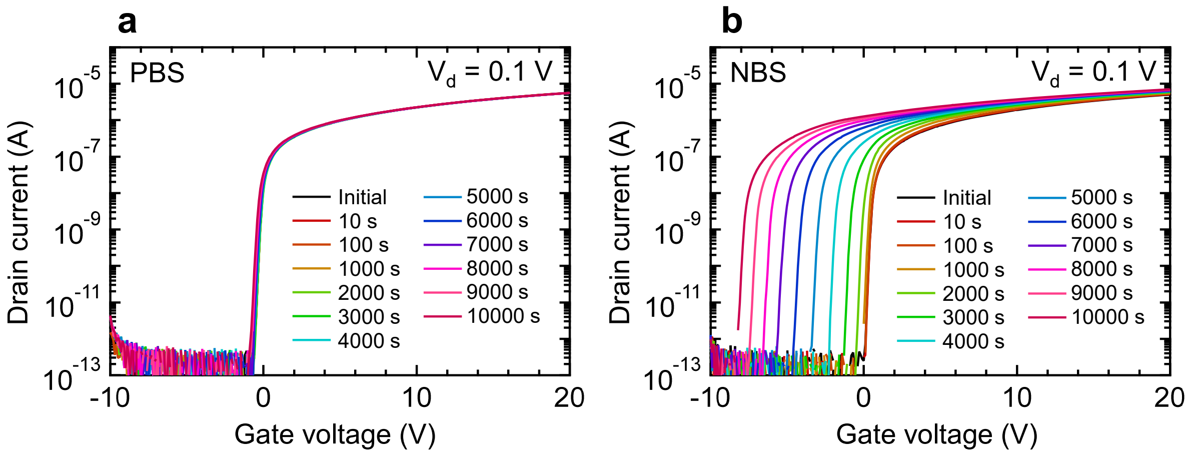

Supplementary Figure 3. Reliability of the In_2O_3:H TFT under a humidity of 70%. Changes in the transfer characteristics of the In_2O_3:H ($R[H_2]$ = 5%) TFT during the (a) PBS and (b) NBS tests. The V_{gs} values under the PBS and NBS tests were +20 and −20 V, respectively.
